# Event and Comment

**Published:** 1923-03

**Authors:** 


### DENTAL REGISTER

THE MAGAZINE OF DENTISTRY

Vol. LXXVII MARCH, 1923 No. 3

### EVENT AND COMMENT

BY VOTRE AMI DENTAIRE

RELATIVITY: *Finite vs. Infinite.*—Possibly a great many of the readers of THE DENTAL REGISTER have read of Mr. Einstein and of his theory of relativity. The scientific theories which are enunciated by great scientists are often based upon a fundamental thought so far removed from what the superficial deductions of the theory are that the real truth of the theory is not often understood. Thus it seems to be with the Einstein theory. When Columbus was trying to convince scientists that the world is round the prevailing theory was that the earth was a flat space. The present idea of space is very similar because it conceives space as rectangular with straight lines. Einstein has applied the spherical theory of Columbus to space and deducts therefrom that space is spherical; that a line of travel through space brings one back to the same place; that there is no infinity, but space is absolutely finite. It is very large of course but still finite. This principle may have many new angles and bring explanation to many scientific problems. It coincides with the wisdom of the ancients that the circle symbolizes the universe and all there is in it. Mr. Einstein has placed his name on a high pinnacle of fame for scientific research.

In Hawaii there is a custom among the natives to pray another to death. It is just the opposite idea practiced among some Americans to pray a sick person back to health. Both these practices illustrate the force of suggestion and demonstrate that the practice is not so idiotic as at first appears. Prayer itself as given to human beings is a force which, together with the above explanation of its suggestive value, can be made very useful. Christians find it useful and often find it needful. The suggestive force of the "movies" is a subject constantly kept in mind by the censors. Many events in the practice of dentistry require a knowledge of the value of suggestion. The expression "Imagination Rules," as used by lecturers on psychology, is well illustrated by the public fear of dental pain. The extraction of a tooth is really a relief and a cessation of the pain, but the patient thinks otherwise and blames the operation for the pain.

---

The fad of having a particular color wax for waxing up plates in prosthetic work is becoming noticeable. Pink and yellow have been the vogue for many years, but this spring a green color is being advocated and also a black and a maroon. Of course a college professor will rightly explain that so far as the waxing up is concerned it makes no difference what the color of the wax is and that is right. It really does not make any difference. Here is where the difference comes in: it is like the lady with neurasthenia who insists on pink pills; the color has a psychologic value and distinguishes one make or brand of wax from another, although all are wax. The color really does not add to the cost either. The purity may, but the color never. Purity would call of lack of artificial color. It is something like tea. It was started plain, but someone took up the plan of coloring it, and it really has taken such a hold, that most of us, if we saw real tea, would not buy it, but would buy the colored tea. There is something to it. But why start it on wax?

